# Tracing the phylogenetic history of the Crl regulon through the *Bacteria* and *Archaea* genomes

**DOI:** 10.1186/s12864-019-5619-z

**Published:** 2019-04-16

**Authors:** A. Santos-Zavaleta, E. Pérez-Rueda, M. Sánchez-Pérez, D. A. Velázquez-Ramírez, J. Collado-Vides

**Affiliations:** 1 Programa de Genómica Computacional, Centro de Ciencias Genómicas, Universidad Nacional Autónoma de México, 62210 Cuernavaca, Morelos Mexico; 2 Instituto de Investigaciones en Matemáticas Aplicadas y en Sistemas, Sede Mérida, Universidad Nacional Autónoma de México, Unidad Académica de Ciencias y Tecnología, 97302 Mérida, Yucatán Mexico; 3 Centro de Genómica y Bioinformática, Facultad de Ciencias, Universidad Mayor, Santiago, Chile

**Keywords:** Crl regulon, Stress response, Transcription factors, Comparative genomics, Bacteria, Archaea

## Abstract

**Background:**

Crl, identified for curli production, is a small transcription factor that stimulates the association of the σ^S^ factor (RpoS) with the RNA polymerase core through direct and specific interactions, increasing the transcription rate of genes during the transition from exponential to stationary phase at low temperatures, using indole as an effector molecule. The lack of a comprehensive collection of information on the Crl regulon makes it difficult to identify a dominant function of Crl and to generate any hypotheses concerning its taxonomical distribution in archaeal and bacterial organisms.

**Results:**

In this work, based on a systematic literature review, we identified the first comprehensive dataset of 86 genes under the control of Crl in the bacterium *Escherichia coli* K-12; those genes correspond to 40% of the σ^S^ regulon in this bacterium. Based on an analysis of orthologs in 18 archaeal and 69 bacterial taxonomical divisions and using *E. coli* K-12 as a framework, we suggest three main events that resulted in this regulon’s actual form: (i) in a first step, *rpoS*, a gene widely distributed in bacteria and archaea cellular domains, was recruited to regulate genes involved in ancient metabolic processes, such as those associated with glycolysis and the tricarboxylic acid cycle; (ii) in a second step, the regulon recruited those genes involved in metabolic processes, which are mainly taxonomically constrained to *Proteobacteria*, with some secondary losses, such as those genes involved in responses to stress or starvation and cell adhesion, among others; and (iii) in a posterior step, Crl might have been recruited in *Enterobacteriaceae*; because its taxonomical pattern constrained to this bacterial order, however further analysis are necessary.

**Conclusions:**

Therefore, we suggest that the regulon Crl is highly flexible for phenotypic adaptation, probably as consequence of the diverse growth environments associated with all organisms in which members of this regulatory network are present.

**Electronic supplementary material:**

The online version of this article (10.1186/s12864-019-5619-z) contains supplementary material, which is available to authorized users.

## Background

Gene expression in bacteria is coordinated through the DNA-binding transcription factors (TFs), blocking or allowing the access of the RNA polymerase (RNAP)-sigma factor to the promoter and providing bacteria with the ability to activate or repress multiple genes under different metabolic stimuli or growth conditions. In the bacterium *Escherichia coli* K-12, seven sigma factors have been experimentally identified, together with around 300 different TFs responsible for recognizing and activating almost all of their genes [1]. Among these, RpoD, or σ^70^, regulates around 40% of the total gene repertoire, whereas alternative sigma factors such as RpoS (σ^S^), the master regulator of the stationary-phase response [2], regulate between 5 and 10% of the total genes in *E. coli* K-12 [3].

Sigma factors and TFs regulate a large diversity of genes, hierarchically organized in regulons [4]. Previous comparative genomics studies have suggested that regulons exhibit considerable plasticity across the evolution of bacterial species [5]. In this regard, comparison of the gene composition of the PhoPQ regulon in *E. coli* and *Salmonella enterica* serovar Typhimurium revealed a very small overlap in both species, suggesting a low similarity in composition between the target genes that are regulated by PhoP in *S.* Typhimurium strains and in *E. coli* K-12 [6]. Incidentally, this plasticity in bacterial regulons is evidence of lineage-specific modifications [7].

We conducted an exhaustive analysis concerning the conservation of the Crl regulon in Bacteria and Archaea cellular domains, using as a reference the currently known system in *E. coli* K-12. Contrary to the most common regulatory mechanisms that involve the direct binding to operators or activators, Crl is an RNAP holoenzyme assembly factor that was originally identified in curli production. It is expressed at low temperatures (30 °C) [8] during the transition phase between the exponential and stationary phases, under low osmolarity, as well as in stationary phase [9]. In *E. coli*, Crl has a global regulatory effect in stationary phase, through σ^S^, as it reorganizes the transcriptional machinery [10], stimulating the association of σ^S^ with the RNAP core, tilting the competition between σ^S^ and σ^70^ during the stationary phase in response to different stress conditions [11, 12] [8, 13]; its production is concomitant with the accumulation of σ^S^ [8].

Assembling the different pieces of the Crl regulon and its regulatory network into one global picture is one of our objectives in this work. The evaluation of this regulon in *Bacteria* and *Archaea* will provide clues about how the regulation of genes by Crl has been recruited in all the organisms, i.e., if the regulated genes were recruited similar to Crl or if they followed different pathways. To this end, 86 genes under the control of Crl in *E. coli* K-12 were compiled from exhaustive literature searches. To our knowledge, this is the first attempt to describe the genes regulated by Crl in *E. coli* K-12; in addition, few Crl homologs were identified among bacterial and archaeal genomes, constrained to *Enterobacteriaceae* species. Finally, members of the regulon were identified as widely distributed beyond enterobacteria, suggesting that Crl was recruited in a secondary evolutionary event to regulate a specific subset of genes, most likely genes involved in a functional response in enterobacteria to contend against starvation.

## Results

### 86 genes belong to the Crl regulon

**Fig. 1:**
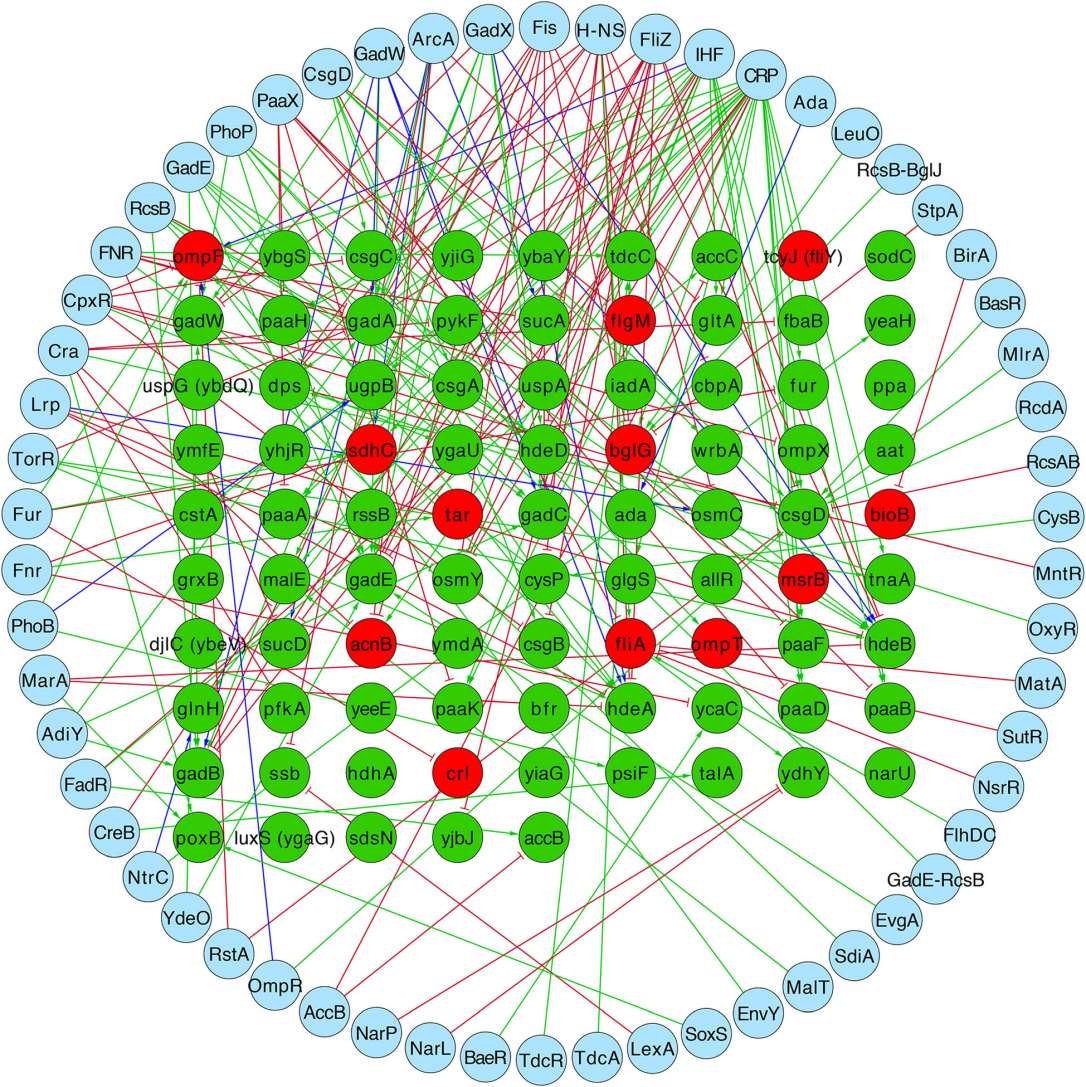
Crl regulatory network in *E. coli* K-12. In green are those genes regulated positively and in red those regulated negatively. The regulatory effects of additional TFs are shown as green solid lines for activation and red solid lines for repression

Available information regarding the Crl regulon was gathered through an exhaustive review of the literature. In this regard, diverse experimental evidences were considered significant for determining the association between the regulated genes and Crl protein regulator, such as gene expression analysis (transcriptional fusions), mapping of signal intensities (RNA-seq or microarray analysis), and inferences made from a mutant phenotype (mutation of a TF with a visible cell phenotype), among other analyses. Therefore, 86 genes were included in this work as members of the σS sigmulon, of which 37 had already been reported in both RegulonDB and EcoCyc database; whereas, 49 genes identified by microarray data and *crl rpoS* double mutants [8–16], in previous works were also added (see Additional file 1). From the 86 genes identified as members of this regulon (see Table 1 and Fig. 1), 34 have a σ^S^-type promoter experimentally determined and 8 genes have 13 σ^S^-type promoters predicted by computational approaches [17]. These 86 genes are organized in 77 transcription units (TUs), where 52% are TUs with only one gene.

**Table 1:** Genes regulated by Crl in *Escherichia coli*

Gene	Bnumber	TU(s)	TFs	Effect of Crl	Evidence	Reference(s)	GO Terms
*aat*	b0885	*aat*		+	GEA and IMP	[11]	protein catabolic process, ubiquitin-dependent protein catabolic process via the N-end rule pathway
*accB*	b3255	*accBC*	AccB (−), FadR(+)	+	GEA and IMP	[11]	lipid metabolic process, fatty acid metabolic process, fatty acid biosynthetic process
*accC*	b3256	*accBC*	AccB (−), FadR(+)	+	GEA and IMP	[11]	lipid metabolic process, fatty acid metabolic process, fatty acid biosynthetic process, metabolic process, negative regulation of fatty acid biosynthetic process, malonyl-CoA biosynthetic process
*acnB*	b0118	*acnB*	CRP(+) ArcA(−), Cra(−), Fis (−)	–	IMP	[13]	regulation of translation, propionate catabolic process, 2-methylcitrate cycle, glyoxylate cycle, tricarboxylic acid cycle metabolic process
*ada*	b2213	*ada-* ***alkB***	Ada(+/−)	+	GEA	[13]	DNA dealkylation involved in DNA repair, regulation of transcription, cellular response to DNA damage stimulus, metabolic process, methylation, DNA demethylation
*allR*	b0506	*allR*		+	MSI	[10]	regulation of transcription, cellular response to DNA damage stimulus, negative regulation of transcription
*bfr*	b3336	***bfd*** *-bfr*		+	MSI, IMP	[40]^MSI^, [11]^IMP^	iron ion transport, cellular iron ion homeostasis, intracellular sequestering of iron ion, oxidation-reduction process
*bglG*	b3723	*bglG* *bglG* ***FB***	CRP (+), Fis (−), H-NS (−), LeuO (+), RcsB-BglJ (+), StpA (−)	–	MSI, IMP	[16]	regulation of transcription, positive regulation of transcription
*bioB*	b0775	*bioB* ***FCD***	BirA (−)	–	IMP	[13]	biotin biosynthetic process
*cbpA*	b1000	*cbpA* ***M***	Fis (−)	+	GEA	[13]	protein folding
*crl*	b0240	*crl*	Fur (−)	–	MSI, IMP	[12]	regulation of transcription, DNA-templated, cellular protein complex assembly, positive regulation of transcription
*csgA*	b1042	*csgBAC*	CpxR (−), CsgD (+), FliZ (−)	+	APPH, MSI, IMP, GEA, IMP	[8] ^APPH, MSI, IMP^, [9] ^GEA, IMP^	cell adhesion, single-species biofilm formation, amyloid fibril formation
*csgB*	b1041	*csgBAC*	CpxR(−), CsgD(+), FliZ(−)	+	APPH, MSI, IMP, GEA, IMP	[8] ^APPH, MSI, IMP^, [9] ^GEA, IMP^	cell adhesion, single-species biofilm formation, amyloid fibril formation
*csgC*	b1043	*csgBAC*	CpxR (−), CsgD (+), FliZ (−)	+	MSI	[10]	
*csgD*	b1040	*csgD* ***EFG***	BasR (+), Cra (+), CRP (+), CsgD (+), IHF (+), MlrA (+), OmpR (+), RcdA (+), CpxR(−), FliZ (−), RcsAB (−), RstA (−)	+	IMP	[13]	regulation of single-species biofilm formation
*cstA*	b0598	*cstA*	CRP (+)	+	GEA, IMP	[11]	cellular response to starvation
*cysP*	b2425	*cysP* ***UWAM***	CysB (+), H-NS (−)	+	MSI	[10]	sulfur compound metabolic process, transport, sulfate transport, sulfate transmembrane transport
*djlC (ybeV)*	b0649	***ybeU*** *-djlC*		+	MSI	[10]	positive regulation of ATPase activity
*dps*	b0812	*dps*	Fis(−), H-NS(−),IHF(+), MntR(−), OxyR(+)	+	GEA, IMP	[11]	cellular iron ion homeostasis, response to stress, chromosome condensation, response to starvation, oxidation-reduction process
*fbaB*	b2097	*fbaB*	Cra(−)	+	GEA, IMP	[11]	glycolytic process, transcription
*flgM*	b1071	*flgM****N***, *flg* ***A*** *M* ***N***	CsgD(−)	–	GEA, IMP	[13]	regulation of transcription, bacterial-type flagellum organization, negative regulation of proteolysis, negative regulation of transcription
*fliA*	b1922	*fliA* ***Z*** *-tcyJ*	H-NS(+), MatA(−), SutR(−), NsrR(−), CsgD(−), FlhDC(+)	–	IMP	[13]	transcription initiation from bacterial-type RNAP promoter, sporulation resulting in formation of a cellular spore
*fur*	b0683	*fur* ***fldA-uof*** *-fur* ***uof*** *-fur*	CRP(+), Fur(−)	+	MSI, IMP	[12]	regulation of transcription, negative regulation of transcription
*gadA*	b3517	*gadA* ***X***	AdiY(+), ArcA(+), CRP(−), FNR(−), Fis(−), GadE-RcsB(+), GadW(+−), GadX(+), H-NS(−), RcsB(−), TorR(−)	+	MSI	[10]	glutamate metabolic process, carboxylic acid metabolic process, intracellular pH elevation
*gadB*	b1493	*gadBC*	AdiY(+),CRP(−), Fis(−), FliZ(−), GadE(+), GadW(+−), GadX(+), RcsB(+)	+	MSI, GEA	[40]^MSI^, [13]^GEA^	glutamate metabolic process, carboxylic acid metabolic process, intracellular pH elevation
*gadC*	b1492	*gadBC*	AdiY(+), CRP(−), Fis(−), FliZ(−), GadE(+), GadW(+−), GadX(+), RcsB(+)	+	MSI	[10]	amino acid transmembrane transport, transport, amino acid transport, intracellular pH elevation
*gadE*	b3512	*gadE-* ***mdtEF*** *gadE*	ArcA(+), CRP(−), EvgA(+), FliZ(−), GadE(+), GadW(+), GadX(+), H-NS(−), PhoP(+), YdeO(+)	+	MSI	[10]	regulation of transcription
*gadW*	b3515	*gadW*	GadW (+), GadX (−), H-NS(−), PhoP (+), SdiA (+), YdeO (+)	+	MSI	[10]	regulation of transcription, cellular response to DNA damage stimulus
*glgS*	b3049	*glgS*	CRP(+)	+	GEA	[13]	glycogen biosynthetic process, positive regulation of cellular carbohydrate metabolic process, negative regulation of single-species biofilm formation on inanimate substrate, negative regulation of bacterial-type flagellum-dependent cell motility
*glnH*	b0811	*glnH* ***PQ***	IHF(+), NtrC(+/−)	+	GEA, IMP	[11]	transport, amino acid transport
*gltA*	b0720	*gltA*	ArcA(−), CRP(+), IHF(+)	+	GEA, IMP	[11]	tricarboxylic acid cycle, metabolic process, cellular carbohydrate metabolic process
*grxB*	b1064	*grxB*		+	GEA, IMP	[11]	cell redox homeostasis, oxidation-reduction process
*hdeA*	b3510	*hdeAB-* ***yhiD***	FliZ(−), GadE(+), GadW(+/−), GadX(+/−), H-NS(−), Lrp(−), MarA(−), PhoP(+), RcsB(+), TorR(+)	+	MSI, GEA	[40]^MSI^, [13]^GEA^	cellular response to stress, cellular response to acidic pH
*hdeB*	b3509	*hdeAB-* ***yhiD***	FliZ(−), GadE(+), GadW(+/−), GadX(+/−), H-NS(−), Lrp(−), MarA(−), PhoP(+), RcsB(+), TorR(+)	+	MSI	[10]	response to pH change, cellular response to stress
*hdeD*	b3511	*hdeD*	GadE(+), GadX(+), H-NS(−), PhoP(+),RcsB(+)	+	MSI	[10]	response to pH change
*hdhA*	b1619	*hdhA*		+	GEA, IMP	[11]	lipid metabolic process, metabolic process, steroid metabolic process, lipid catabolic process, bile acid, catabolic process, protein homotetramerization, oxidation-reduction process
*iadA*	b4328	***yjiH*** *G-iadA*		+	MSI	[10]	proteolysis
*luxS (ygaG)*	b2687	*luxS*		+	MSI, GEA, IMP	[10]^MSI^, [11] ^GEA, IMP^	cell-cell signaling involved in quorum sensing, L-methionine biosynthetic process from S-adenosylmethionine, quorum sensing
*malE*	b4034	*malE* ***FG***	CRP(+), CreB(−), Fis(+), MalT(+)	+	GEA, IMP	[11]	cellular response to DNA damage stimulus, carbohydrate transport, maltose transport, detection of maltose stimulus, maltodextrin transport, cell chemotaxis
*msrB*	b1778	*msrB*		–	IMP	[13]	protein repair, response to oxidative stress
*narU*	b1469	*narU*		+	MSI	[10]	nitrate transport, nitrite transport, nitrate assimilation
*ompF*	b0929	*ompF*	CRP(+), CpxR(−), EnvY(+), Fur(+), IHF(+/−), OmpR(+/−), PhoB(+), RstA(−)	–	GEA, IMP	[41]	transport, ion transport, ion transport, drug transmembrane transport, bacteriocin transport
*ompT*	b0565	*ompT* ***envY*** *-ompT*	PhoP(+)	–	IMP	[13]	proteolysis
*ompX*	b1482	*ompX*	FNR(−)	+	GEA, IMP	[11]	
*osmC*	b4376	*osmC*	H-NS(−), Lrp(+/−)	+	GEA, IMP	[11]	hyperosmotic response, response to oxidative stress, response to hydroperoxide, oxidation-reduction process
*osmY*	b1388	*osmY*	CRP(−), Fis(−), FliZ(−), IHF(−), Lrp(−)	+	GEA, IMP	[11]	response to osmotic stress
*paaA*	b1389	*paaAB* ***C*** *D* ***E*** *F* ***G*** *H* ***IJ*** *K*	CRP(+), IHF(+), PaaX(−)	+	MSI	[10]	phenylacetate catabolic process, oxidation-reduction process
*paaB*	b1391	*paaAB* ***C*** *D* ***E*** *F* ***G*** *H* ***IJ*** *K*	CRP (+), IHF (+), PaaX (−)	+	MSI	[10]	phenylacetate catabolic process
*paaD*	b1393	*paaAB* ***C*** *D* ***E*** *F* ***G*** *H* ***IJ*** *K*	CRP (+), IHF (+), PaaX (−)	+	MSI	[10]	phenylacetate catabolic process
*paaF*	b1395	*paaAB* ***C*** *D* ***E*** *F* ***G*** *H* ***IJ*** *K*	CRP (+), IHF (+), PaaX (−)	+	MSI	[10]	lipid metabolic process, fatty acid metabolic process, phenylacetate catabolic process
*paaH*	b1398	*paaAB* ***C*** *D* ***E*** *F* ***G*** *H* ***IJ*** *K*	CRP(+), IHF(+), PaaX(−)	+	MSI	[10]	fatty acid metabolic process, phenylacetate catabolic process, oxidation-reduction process
*paaK*	b3916	*paaAB* ***C*** *D* ***E*** *F* ***G*** *H* ***IJ*** *K*	CRP(+), IHF(+), PaaX(−)	+	MSI	[10]	metabolic process, phenylacetate catabolic process
*pfkA*	b0871	*pfkA*	Cra(−)	+	GEA, IMP	[11]	fructose 6-phosphate metabolic process, glycolytic process
*poxB*	b4226	*poxB,* *poxB-l* ***taE-ybjT***	Cra(+), MarA(+), SoxS (+)	+	GEA, IMP	[11]	pyruvate metabolic process, oxidation-reduction process
*ppa*	b0384	*ppa*		+	GEA, IMP	[11]	phosphate-containing compound metabolic process
*psiF*	b1676	***phoA*** *-psiF*	PhoB(+)	+	MSI	[10]	
*pykF*	b1235	*pykF*	Cra(−)	+	GEA, IMP	[11]	glycolytic process, metabolic process, response to heat, phosphorylation
*rssB*	b0721	*rssB*		+	IMP	[10]	protein destabilization, positive regulation of proteolysis, regulation of nucleic acid-templated transcription (phosphorelay signal transduction system)
*sdhC*	b4719	*sdhC* ***DAB*** *-sucA* ***BC*** *D*	CRP(+), Fur(+), ArcA(+/−), Fnr(−)	–	IMP	[13]	aerobic respiration. Cytochrome complex assembly, tricarboxylic acid cycle, oxidation-reduction process
*sdsN*	b1646	*sdsN*		+	GEA	[42]	small RNA
*sodC*	b4059	*sodC*		+	GEA	[13]	superoxide metabolic process, removal of superoxide radicals, oxidation-reduction process
*ssb*	b0726	*ssb*	ArcA(−), LexA(−)	+	GEA, IMP	[11]	recombinational repair, DNA replication, cellular response to DNA damage stimulus, SOS response
*sucA*	b0729	*sucA* ***B*** *sucA* ***BC*** *D*	ArcA(+/−), FNR(−), IHF(−)	+	GEA, IMP	[11]	glycolytic process, tricarboxylic acid cycle, metabolic process, oxidation-reduction process
*sucD*	b2464	*sucA* ***B*** *sucA* ***BC*** *D*	ArcA(+/−),FNR(−),IHF(−)	+	GEA, IMP	[11]	tricarboxylic acid cycle, metabolic process, protein autophosphorylation
*talA*	b1886	*talA-* ***tktB***	CreB(+)	+	GEA, IMP	[11]	carbohydrate metabolic process, pentose-phosphate shunt
*tar*	b1920	*tar-* ***tap-cheRBYZ***	Fnr(+)	–	IMP	[13]	chemotaxis, signal transduction
*tcyJ (fliY)*	b3116	*tcyJ* *fliA* ***Z*** *-tcyJ*	H-NS(+), MatA(−), SutR(−), NsrR(−), CsgD(−), FlhDC(+)	–	IMP	[13]	L-cystine transport
*tdcC*	b3708	*tdc* ***AB*** *C* ***DEFG*** *, tdc* ***B*** *C* ***DEFG***	CRP(+), FNR(+), IHF(+), TdcA(+), TdcR (+)	+	MSI	[10]	L-serine transport, threonine transport, proton transport, serine transport
*tnaA*	b3453	*tna* ***C*** *A* **B**	CRP(+), TorR (+)	+	GEA, IMP	[11]	cellular amino acid metabolic process, aromatic amino acid family metabolic process
*ugpB*	b3495	*ugpB* ***AECQ***	CRP(+), PhoB(+/−)	+	GEA, IMP	[11]	glycerophosphodiester transport, transport, glycerol-3-phosphate transport
*uspA*	b0607	*uspA*	FadR(−), IHF(+)	+	GEA, IMP	[11]	response to stress
*uspG (ybdQ)*	b1004	*uspG*		+	GEA, IMP	[11]	response to stress, protein adenylylation, protein autophosphorylation, nucleotide phosphorylation, regulation of cell motility
*wrbA*	b0453	*wrbA-* ***yccJ***	CsgD(+)	+	MSI, GEA, IMP	[13]^MSI^, [11] ^GEA, IMP^	response to oxidative stress, negative regulation of transcription
*ybaY*	b0753	*ybaY*		+	MSI	[10]	
*ybgS*	b0897	*ybgS*		+	MSI	[10]	
*ycaC*	b1674	*ycaC*	BaeR(+), Fnr(−)	+	MSI, GEA, IMP	[10]^MSI^, [11] ^GEA, IMP^	metabolic process
*ydhY*	b1784	*ydhY* ***VWXUT***	FNR (+), NarL (−), NarP (−)	+	MSI	[10]	oxidation-reduction process
*yeaH*	b2013	*yea* ***G*** *H*	NtrC (+)	+	MSI	[10]	
*yeeE*	b2665	*yeeE* ***D***		+	MSI	[10]	
*ygaU*	b3535	*ygaU*	CpxR (+)	+	GEA, IMP	[11]	
*yhjR*	b3555	*yhjR*		+	MSI	[10]	bacterial cellulose biosynthetic process
*yiaG*	b4045	*yiaG*		+	MSI	[10]	regulation of transcription
*yjbJ*	b4329	*yjbJ*	FliZ (−)	+	MSI	[10]	
*yjiG*	b1044	***yjiH*** *G-iadA*		+	MSI	[10]	
*ymdA*	b1138	*ymdA*		+	MSI	[10]	
*ymfE*	b0885	*ymfED*		+	MSI	[10]	

Genes regulated by Crl, Bnumbers, TUs to which they belong (in bold are possible candidates regulated by Crl, since they are controlled by Crl and σ^S^, but they did not have a change of expression in the data we evaluated), TFs regulating the TU, the effect of Crl, evidences, references, and associated GO terms. Experimental evidence types supporting regulation by Crl: *APPH* Assay of protein purified to homogeneity, *GEA* Gene expression analysis, transcriptional fusions (*lacZ*), *MSI* Mapping of signal intensities, such as RNA-seq or microarray analysis; *IMP* Inferred from mutant phenotype (such as a mutation of a TF that has a visible cell phenotype and it is inferred that the regulator might be regulating the genes responsible for the phenotype). Growth conditions were 30 °C, as the stationary phase was induced for all experiments. All experiments were done with *E. coli* K-12 or derivative strains. This information, including regulatory interactions can be accessed at RegulonDB (http://regulondb.ccg.unam.mx/) by consulting the Crl regulon

**Fig. 2:**
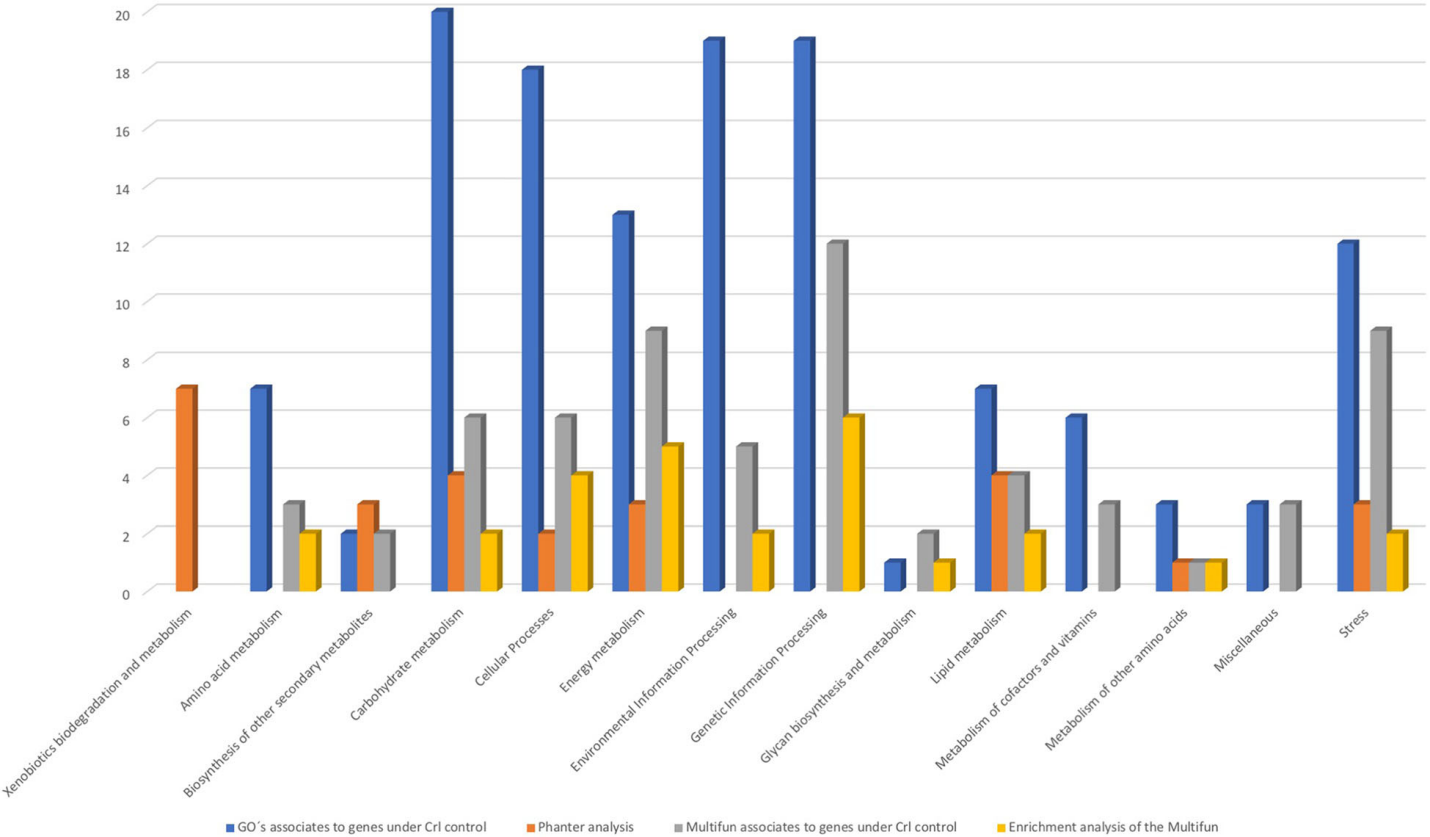
Functions associated with genes under the control of Crl. GOs and Multifun-associated genes under Crl control and enrichment analysis with the PANTHER classification system and Multifun. Categories of KEGG used to classify GOs and Multifun terms are shown on the X-axis, and the number of GOs associated with each category are shown on the Y-axis

Previously, genes under the control of Crl were classified in four main categories depending on their role(s) in the cell: DNA metabolism, central metabolism, response to environmental modifications, and miscellaneous [11]. Based on Gene Ontology (GO) annotations, multifunctional classification, and KEGG pathway maps to categorize functions, Crl-regulated genes appear to be involved in metabolic processes such as energy metabolism, amino acid, carbohydrate, and lipid metabolism, and biosynthetic processes such as glycan biosynthesis and biosynthesis of other secondary metabolites, among other metabolic processes. These functions correlate with results of the enrichment analysis using PANTHER, which showed that catabolic processes, metabolic processes, and cellular responses to xenobiotic stimuli were overrepresented among the functions associated with genes under the control of Crl (See Fig. 2).

In general, genes under Crl control are involved in regulating many aspects of cellular metabolism through Crl’s interaction with a subset of genes of the σ^S^ regulon [8] in addition to quorum sensing playing a major role in cell-to-cell communication during stationary phase and in different processes such as biofilm formation or virulence, transporters [11] and genes involved in the uptake and utilization of β-glucosides [16].

### Composition of the Crl regulon

In order to determine whether additional TFs also regulate the genes under the control of Crl, RegulonDB was used to evaluate how genes associated with Crl are also regulated by alternative TFs or sigma factors. A total of 24 genes were identified as exclusively controlled by Crl, whereas 62 are regulated by additional TFs (See Additional file 2). In this regard, 55 different TFs are involved in the regulation of genes associated with Crl, including Crp, IHF, H-NS, Fis, FNR, ArcA, GadX, GadW, GadE, and CsgD (Table 1), suggesting that all genes regulated by Crl are also involved in multiple functions beyond the stationary phase, or, alternatively, phase transition has to regulate genes involved in large number of different functions. It is interesting that six of seven global regulators identified in the regulatory network of *E. coli* are also associated with the set regulated by Crl. Another way to look at this small network is that 19 genes of the total of Crl-regulated genes are regulated by one TF, 11 by two TFs, and 14 by three different TFs. Therefore, Crl is regulating positively 73 (85%) genes, whereas 12 (15%) genes are regulated negatively (Table 1). The predominance of positive regulation suggests that genes associated with this regulon are in high demand according the demand theory suggested by Savageau [18], and the activities of their proteins are enhanced to contend with varied environmental stimuli. Thirty-four of the 86 genes have a σ^S^-type promoter that was experimentally determined (RegulonDB). Finally, the promoters of 49 genes identified as members of Crl and of the σ^S^ sigmulon, based on transcriptional fusions and microarray analysis data, remain to be experimentally determined.

### Phylogenetic analysis of Crl

**Fig. 3:**
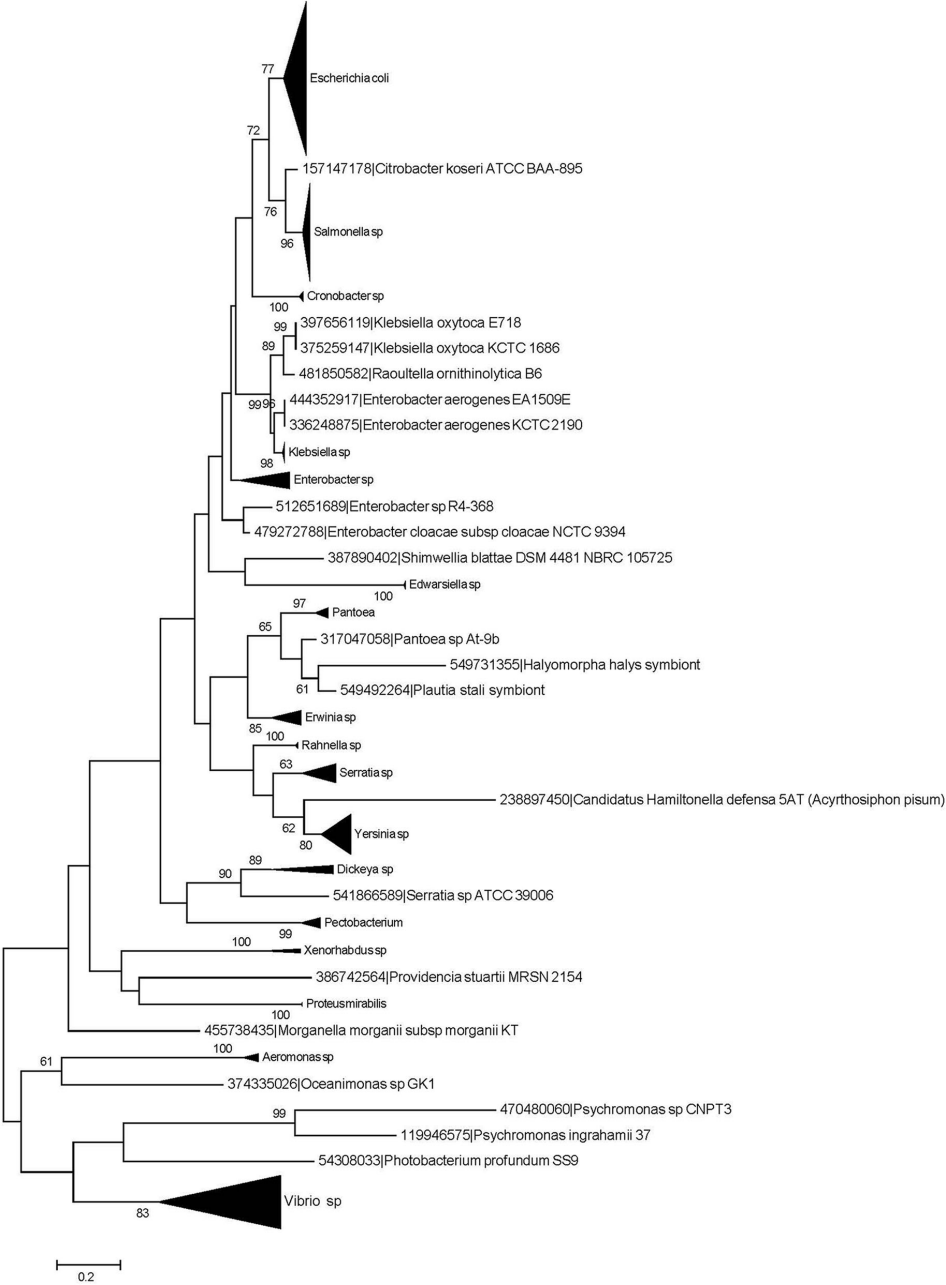
Phylogenetic tree of Crl. Phylogenetic tree based on Crl of *E. coli* and homologs in other organisms generated via maximum likelihood analysis, with 1000 replicates. Species with bootstrap values higher than 60% are displayed. The black triangles to the right of the branches indicate multiple species for those genera

In order to evaluate the phylogenetic history of Crl across the bacterial and archaeal cellular domains, its homologs were identified as described in the Methods section, and a phylogenetic tree with maximum likelihood was generated (Fig. 3 and Additional file 3). From this analysis, we found that Crl and its homologs are distributed almost exclusively among *Gammaproteobacteria* but do not share homology with proteins from other taxonomical divisions, as has been previously noted for *E. coli*, *Vibrio* spp., *Citrobacter* spp., *Salmonella* spp., and *Enterobacter aerogenes* [16]. Additional information suggests that Crl is less widespread and less conserved at the sequence level than σ^S^ [19]. In this regard, four conserved residues (Y22, F53, W56, and W82) are important for Crl activity and for Crl-σ^S^ interaction but not for Crl stability in *S*. *Typhimurium* [19]. On one hand it is probable that Crl homologs exist in some σ^S^-containing bacteria; however, some species might use alternative strategies to favor σ^S^ interaction with the core of the RNAP [19]. Therefore, our phylogenetic analysis suggests that Crl is a protein conserved and constrained to *Gammaproteobacteria*, such as in *Vibrio* spp., *Klebsiella* spp., *Enterobacter* spp., and *Escherichia coli*. Contrary to Crl, several other of the TFs that co-regulate the Crl regulon, are present beyond the gamma-proteobacterial, probably pre-dating regulation of some of the target genes, which have been more recently subject to Crl regulation.

In addition, homologous of Crl were found in low copy numbers, i.e., one Crl-like protein per genome. This information, together with the distribution of σ^S^, suggests that the regulator was recruited as an element to regulate a subset of σ^S^-regulated genes in *Gammaproteobacteria*. In this regard, it is interesting that genes under the control of Crl contain an UP element with A/T-rich DNA sequence upstream of a poorly conserved − 35 promoter which may serve for alpha subunit binding of RNAP; suggesting that Crl could play a fundamental role in the contacts between RNAP and its promoter [13]. In addition, Crl would increase transcription rate during the transition from growing to stationary phase at low temperatures [8], using indole as an effector molecule. In summary, this result opens the question explored in the follow section of whether genes regulated by Crl are also constrained to this taxonomical division.

### Taxonomical distribution of Crl-regulated genes

**Fig. 4:**
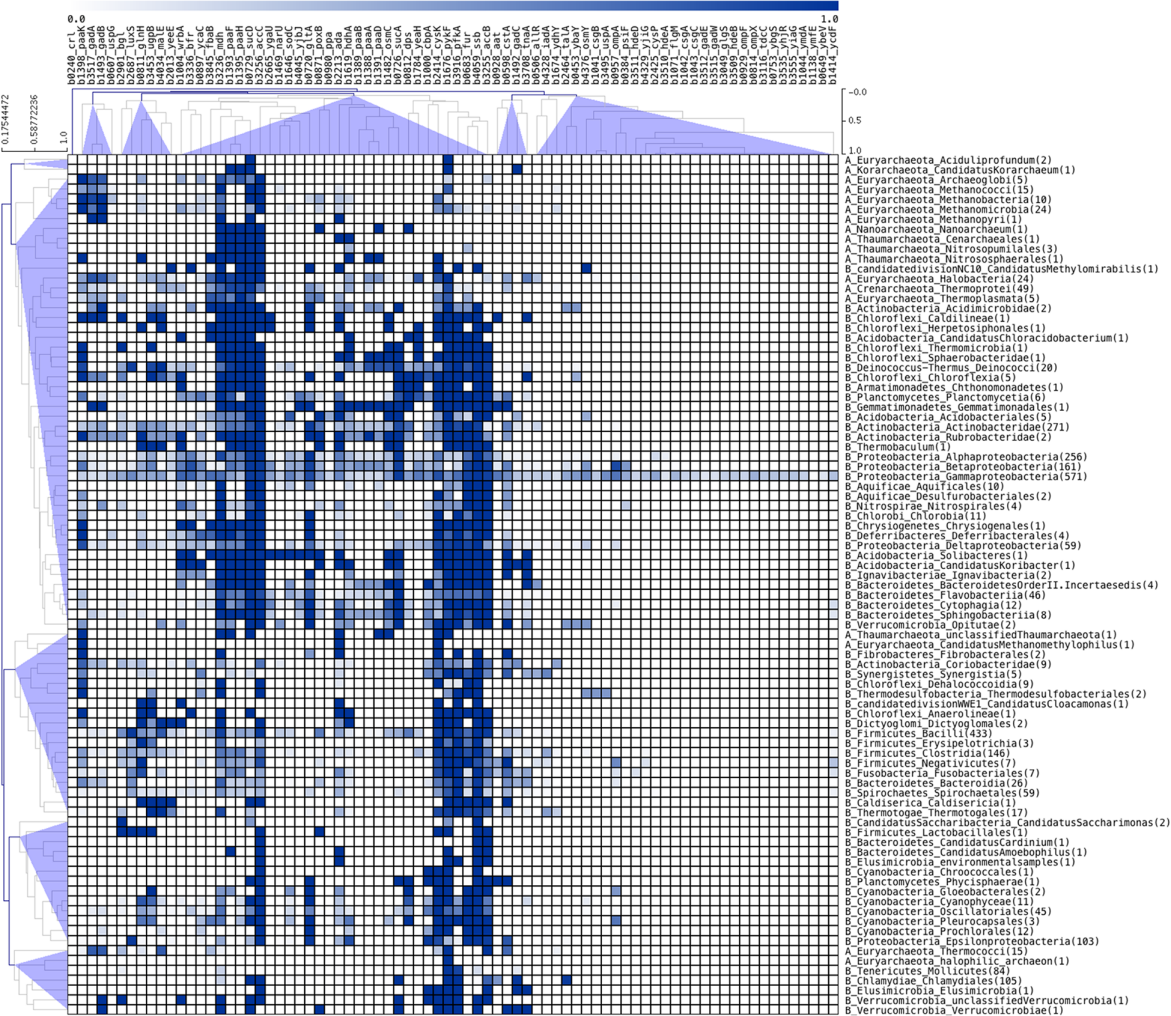
Taxonomic distribution of orthologs from the perspective of *E. coli* K-12. A single linkage-clustering algorithm with no leaf order optimization was applied with Pearson distance as the similarity measure. The display clustering results were obtained using the MeV program [39]. The conserved groups across the different taxonomic groups are indicated. Each column denotes Crl-regulated genes, whereas rows denote taxonomic groups. The bar at the top of the figure indicates the relative abundance of orthologs per group, represented as a percentage, where a value of 1 corresponds to 100% presence and 0% indicates a division without any ortholog of the Crl regulon in the taxonomic group

Based on the identification of orthologs of 86 Crl-regulated genes, we evaluated their taxonomical distribution across archaea and bacteria sequence genomes, as described in Methods (See Fig. 4 and Additional file 4). Based on a taxonomical profile, we determined that the evolution of the Crl regulon seems to have involved diverse losses and gains of regulatory interactions. It is possible that large portions of the regulatory network associated with Crl evolved through extensive genetic changes during the evolution of the species studied. Indeed, we suggest three main events modeled the evolution of this regulon: (i) the regulation of a large number of genes widely distributed among *Bacteria* and *Archaea*, such as those genes involved in ancient metabolic processes such as glycolysis (*fbaB*, *pykF*, *pfkA,* and *sucA*) and those involved in the tricarboxylic acid cycle (*gltA* and *sucD*) [20]; (ii) the regulation of genes with a distribution pattern mainly constrained to *Proteobacteria*, with some secondary losses in other organisms, such as those genes involved in response to stress and starvation (*cstA* and *hdcA*) or cell adhesion (*csgA* and *csgB*), among others; and (iii) the recruitment of Crl as a consequence of its emergence in *Enterobacteriales*. It is interesting that Crl-regulated genes are also part of the σ^S^ sigmulon, where there are no essential genes [21–24]. All these elements suggest that the Crl regulon is highly flexible for phenotypic adaptation, probably as a consequence of the diverse growth environments associated with the organisms in which members of this regulatory network are present.

## Conclusions

Crl stimulates -but can also repress- the association of σ^S^ with the RNAP core in *E. coli* K-12 through direct and specific interactions, increasing, or decreasing, the transcription rate of a subset of genes of the σ^S^ sigmulon. This TF has been described during the transition to stationary phase at low temperatures. In our work, based on an exhaustive literature search, we found 86 genes under the control of Crl in *E. coli*. We considered that the quality of the experiments could compensate the few number of papers where evidences associated to the *rpoS-crl* and its target genes; such as microarray analysis, mutations, assay with purified proteins, among others; however, a large number of records and sources were evaluated to consider the dataset as significant. Indeed, all interactions reported in this work meet the same criteria to be considered in RegulonDB. These protein-coding genes were retrieved mainly from microarray and mutation analyses, among other experimentally supported evidence. Gathering this regulon offers a wider physiological role than previously assumed for Crl. Certainly, these genes are associated with multiple functions, including xenobiotic processes, biofilm formation, metabolic, catabolic, and biosynthetic processes, responses to different stress conditions, and protein assembly, amino acid transport, and transcriptional processes, among others. The diverse functions regulated by Crl suggest that these genes play a fundamental role in multiple functions to respond to environmental changes, mainly those associated with stationary-phase growth at low temperatures. In addition, we conducted an exhaustive analysis concerning the conservation of the regulon Crl among the Bacteria and Archaea genomes, using as starting point, the knowledge gathered for *E. coli* K-12. From this analysis, Crl was identified in low copy numbers and constrained to the *Enterobacteriales* order, whereas the homologs of all regulated genes were found to be widely distributed beyond enterobacteria, suggesting that Crl was recruited in a secondary event to regulate a specific subset of genes for which the regulation (stimulation or repression) of Crl and σ^S^ helps bacteria in the phase transition.

## Methods

### Identification of Crl-regulated genes

We performed an exhaustive search of the literature related to Crl (GI: 114152792) in *E. coli* K-12 in PubMed [25] under the following search strategy: coli in the title (to exclude spurious articles) and crl, rpoS (ID: NP_417221) and regulation both in title and in all fields in their different combinations (coli[all field] crl[all field] rpos[all field] regulation[all field]; coli[ti] rpoS[ti] regulation[ti]; coli[ti] crl[all field] rpos[all field] regulation[all field]; coli[ti] crl[ti] regulation[all field]; coli[ti] rpoS[ti] crl[all field] regulation[all field]; coli[ti] rpoS[ti] crl[ti] regulation[all field]). Fifty nine unique articles with different search profiles were obtained and exhaustively revised, from which, 10 were selected since they contained information on regulatory interactions in *E. coli* on *rpoS-crl* and its target genes, such as microarray analysis, mutations, and assay with purified proteins, among others (Table 1). Finally, we searched for gene/operon notes in RegulonDB and EcoCyc [3, 26] for Crl interactions and σ^S^ promoters, for assembling the network of regulation of Crl.

The regulatory network generated was displayed using the Cytoscape program, version 3.3.0 [27], with information obtained in the identified papers as well as information contained in RegulonDB [3]. Genes under Crl control were classified based on Gene Ontology (GO) annotations (http://www.geneontology.org/) using the Gene Association Format (GAF 2.0) as well as the Multifun classification scheme [28]. An enrichment analysis was carried out to find overrepresented annotations, using the PANTHER Classification system program, version 12.0; selecting biological processes and *E. coli* as parameters [29, 30]. In addition, we used KEGG to categorize the functions of GOs (http://www.genome.jp/kegg-bin/show_organism?menu_type=pathway_maps&org=eco) [31].

### Identification of Crl homologs

The Crl protein sequence of *E. coli* K-12 (ID: 114152792) was used as the seed to scan all the bacterial and archaeal genomes via a BLASTp search (BLAST version 2.2.30+) [32] (E-value ≤10^− 3^ and coverage ≥60%). All proteins were compared and aligned using the Muscle algorithm [33] with default parameters, and results were manually edited with the program Jalview. Finally, a phylogeny was inferred by the maximum likelihood method with 1000 replicates by using the program MEGA [34] and the Tamura-Nei model.

### Identification of orthologous genes

Orthologous genes have been classically defined as encoding proteins in different species that evolved from a common ancestor via speciation [35] and have retained the same function. In this work, orthologs were identified by searching for bidirectional best hits (BDBHs) in other organisms [36] considering the same conditions as [36] an *E*-value ≤ of 1e− 6; database size fixed (−*z* 5e+ 8), soft filtering of low information content (the −F ‘m S’ option), the Smith–Waterman alignment (−s T), and a coverage of at least 60%.

### Taxonomical distribution of orthologous genes

In order to evaluate the taxonomical distribution of the genes belonging to the Crl regulon, 5321 complete genomes were downloaded from the NCBI’s Refseq genome database [37] and open reading frames (ORFs) that encode predicted proteins were considered. Redundancy was excluded using a web-based tool [38] considering a Genome Similarity Score GCCa≥0.95 [38]**.** In this representative genome dataset, orthologs were traced along 18 archaeal and 69 bacterial cellular divisions. To this end, the relative abundance of the orthologs was calculated as the fraction of genomes in the group that had one ortholog, divided by the total number of genomes per phylum, i.e., the ratio (total number of orthologs in a phylum) / (total number of organisms in phylum). The corresponding matrix was analyzed with a hierarchical complete linkage-clustering algorithm with correlation uncentered as the similarity measure. We used the program MeV to perform the analyses (http://mev.tm4.org/) [39].

## Additional files

Additional file 1: New members of the RpoS-Crl regulon. (DOCX 23 kb)

Additional file 2: Number of regulated genes by different transcription factor in Crl Regulon. (DOCX 15 kb)

Additional file 3: Homologs of Crl in Bacteria and Archaea genomes. (TXT 64 kb)

Additional file 4: Orthologs of Crl regulon in Bacteria and Archaea genomes. (GZ 1446 kb)

## Abbreviations

APPH: Assay of protein purified to homogeneity
BDBHs: Bidirectional best hits
BLASTp: Basic Local Alignment Search Tool (proteins)
Crl: Curling genes regulatory protein
Ecocyc: *E. coli* metabolic database
GAF: cGMP-specific phosphodiesterase, adenylyl cyclase and FhlA domain
GCCa: Genome Similarity Score
GEA: Gene expression analysis, transcriptional fusions (*lacZ*)
GO: Gene onthology
IMP: Inferred from mutant phenotype (such as a mutation of a TF that has a visible cell phenotype and it is inferred that the regulator might be regulating the genes responsible for the phenotype)
Jalview: Java alignment editor
KEGG: Kyoto encyclopedia of genes and genomes database
MEGA: Molecular Evolutionary Genetics Analysis
MSI: Mapping of signal intensities, such as RNA-seq or microarray analysis
Muscle: Multiple sequence comparison by Log-expectation
ORFs: Open reading frames
PANTHER: Protein analysis through evolutionary relationships classification system
PhoPQ: Two-component system of virulence and adaptation
RegulonDB: Regulon database
RNAP: RNA polymerase
RNA-seq: RNA sequencing
RpoD: RNA polymerase sigma factor D
RpoS: RNA polymerase sigma factor S
TFs: Transcription factors
TUs: Transcription units

## References

[1] Perez-Rueda E, Tenorio-Salgado S, Huerta-Saquero A, Balderas-Martinez YI, Moreno-Hagelsieb G (2015). The functional landscape bound to the transcription factors of Escherichia coli K-12. Comput Biol Chem.

[2] Landini P, Egli T, Wolf J, Lacour S (2014). sigmaS, a major player in the response to environmental stresses in Escherichia coli: role, regulation and mechanisms of promoter recognition. Environ Microbiol Rep.

[3] Gama-Castro S, Salgado H, Santos-Zavaleta A, Ledezma-Tejeida D, Muniz-Rascado L, Garcia-Sotelo JS, Alquicira-Hernandez K, Martinez-Flores I, Pannier L, Castro-Mondragon JA (2016). RegulonDB version 9.0: high-level integration of gene regulation, coexpression, motif clustering and beyond. Nucleic Acids Res.

[4] Dufour YS, Kiley PJ, Donohue TJ (2010). Reconstruction of the core and extended regulons of global transcription factors. PLoS Genet.

[5] Lozada-Chavez I, Janga SC, Collado-Vides J (2006). Bacterial regulatory networks are extremely flexible in evolution. Nucleic Acids Res.

[6] Monsieurs P, De Keersmaecker S, Navarre WW, Bader MW, De Smet F, McClelland M, Fang FC, De Moor B, Vanderleyden J, Marchal K (2005). Comparison of the PhoPQ regulon in Escherichia coli and Salmonella typhimurium. J Mol Evol.

[7] Liu R, Ochman H (2007). Origins of flagellar gene operons and secondary flagellar systems. J Bacteriol.

[8] Bougdour A, Lelong C, Geiselmann J (2004). Crl, a low temperature-induced protein in Escherichia coli that binds directly to the stationary phase sigma subunit of RNA polymerase. J Biol Chem.

[9] Arnqvist A, Olsen A, Pfeifer J, Russell DG, Normark S (1992). The Crl protein activates cryptic genes for curli formation and fibronectin binding in Escherichia coli HB101. Mol Microbiol.

[10] Typas A, Barembruch C, Possling A, Hengge R (2007). Stationary phase reorganisation of the Escherichia coli transcription machinery by Crl protein, a fine-tuner of sigmas activity and levels. EMBO J.

[11] Lelong C, Aguiluz K, Luche S, Kuhn L, Garin J, Rabilloud T, Geiselmann J (2007). The Crl-RpoS regulon of Escherichia coli. Mol Cell Proteomics.

[12] Lelong C, Rolland M, Louwagie M, Garin J, Geiselmann J (2007). Mutual regulation of Crl and Fur in Escherichia coli W3110. Mol Cell Proteomics.

[13] Dudin O, Lacour S, Geiselmann J (2013). Expression dynamics of RpoS/Crl-dependent genes in Escherichia coli. Res Microbiol.

[14] Olsen A, Arnqvist A, Hammar M, Sukupolvi S, Normark S (1993). The RpoS sigma factor relieves H-NS-mediated transcriptional repression of csgA, the subunit gene of fibronectin-binding curli in Escherichia coli. Mol Microbiol.

[15] Pratt LA, Silhavy TJ (1996). The response regulator SprE controls the stability of RpoS. Proc Natl Acad Sci U S A.

[16] Schnetz K (2002). Silencing of the Escherichia coli bgl operon by RpoS requires Crl. Microbiology (Reading, England).

[17] Huerta AM, Collado-Vides J (2003). Sigma70 promoters in Escherichia coli: specific transcription in dense regions of overlapping promoter-like signals. J Mol Biol.

[18] Savageau MA (1998). Demand theory of gene regulation. I. Quantitative development of the theory. Genetics.

[19] Monteil V, Kolb A, D'Alayer J, Beguin P, Norel F (2010). Identification of conserved amino acid residues of the Salmonella sigmaS chaperone Crl involved in Crl-sigmaS interactions. J Bacteriol.

[20] Dandekar T, Schuster S, Snel B, Huynen M, Bork P (1999). Pathway alignment: application to the comparative analysis of glycolytic enzymes. Biochem J.

[21] Chen G, Patten CL, Schellhorn HE (2004). Positive selection for loss of RpoS function in Escherichia coli. Mutat Res.

[22] Dong T, Schellhorn HE (2009). Control of RpoS in global gene expression of Escherichia coli in minimal media. Mol Gen Genomics.

[23] Zambrano MM, Siegele DA, Almiron M, Tormo A, Kolter R (1993). Microbial competition: Escherichia coli mutants that take over stationary phase cultures. Science.

[24] Cavaliere P, Norel F (2016). Recent advances in the characterization of Crl, the unconventional activator of the stress sigma factor sigmaS/RpoS. Biomol Concepts.

[25] Geer LY, Marchler-Bauer A, Geer RC, Han L, He J, He S, Liu C, Shi W, Bryant SH (2010). The NCBI BioSystems database. Nucleic Acids Res.

[26] Keseler IM, Mackie A, Santos-Zavaleta A, Billington R, Bonavides-Martinez C, Caspi R, Fulcher C, Gama-Castro S, Kothari A, Krummenacker M (2017). The EcoCyc database: reflecting new knowledge about Escherichia coli K-12. Nucleic Acids Res.

[27] Shannon P, Markiel A, Ozier O, Baliga NS, Wang JT, Ramage D, Amin N, Schwikowski B, Ideker T (2003). Cytoscape: a software environment for integrated models of biomolecular interaction networks. Genome Res.

[28] Serres MH, Riley M (2000). MultiFun, a multifunctional classification scheme for Escherichia coli K-12 gene products. Microb Comp Genomics.

[29] Ashburner M, Ball CA, Blake JA, Botstein D, Butler H, Cherry JM, Davis AP, Dolinski K, Dwight SS, Eppig JT (2000). Gene ontology: tool for the unification of biology. The gene ontology consortium. Nat Genet.

[30] Thomas PD, Campbell MJ, Kejariwal A, Mi H, Karlak B, Daverman R, Diemer K, Muruganujan A, Narechania A (2003). PANTHER: a library of protein families and subfamilies indexed by function. Genome Res.

[31] Kanehisa M, Furumichi M, Tanabe M, Sato Y, Morishima K (2017). KEGG: new perspectives on genomes, pathways, diseases and drugs. Nucleic Acids Res.

[32] Altschul SF, Madden TL, Schaffer AA, Zhang J, Zhang Z, Miller W, Lipman DJ (1997). Gapped BLAST and PSI-BLAST: a new generation of protein database search programs. Nucleic Acids Res.

[33] Edgar RC (2004). MUSCLE: multiple sequence alignment with high accuracy and high throughput. Nucleic Acids Res.

[34] Tamura K, Stecher G, Peterson D, Filipski A, Kumar S (2013). MEGA6: molecular evolutionary genetics analysis version 6.0. Mol Biol Evol.

[35] Fitch WM (1970). Distinguishing homologous from analogous proteins. Syst Zool.

[36] Moreno-Hagelsieb G, Latimer K (2008). Choosing BLAST options for better detection of orthologs as reciprocal best hits. Bioinformatics.

[37] Haft DH, DiCuccio M, Badretdin A, Brover V, Chetvernin V, O'Neill K, Li W, Chitsaz F, Derbyshire MK, Gonzales NR (2018). RefSeq: an update on prokaryotic genome annotation and curation. Nucleic Acids Res.

[38] Moreno-Hagelsieb G, Wang Z, Walsh S, ElSherbiny A (2013). Phylogenomic clustering for selecting non-redundant genomes for comparative genomics. Bioinformatics.

[39] Saeed AI, Bhagabati NK, Braisted JC, Liang W, Sharov V, Howe EA, Li J, Thiagarajan M, White JA, Quackenbush J (2006). TM4 microarray software suite. Methods Enzymol.

[40] Weber H, Polen T, Heuveling J, Wendisch VF, Hengge R (2005). Genome-wide analysis of the general stress response network in Escherichia coli: sigmaS-dependent genes, promoters, and sigma factor selectivity. J Bacteriol.

[41] Pratt LA, Silhavy TJ (1998). Crl stimulates RpoS activity during stationary phase. Mol Microbiol.

[42] Hao Y, Updegrove TB, Livingston NN, Storz G (2016). Protection against deleterious nitrogen compounds: role of sigmaS-dependent small RNAs encoded adjacent to sdiA. Nucleic Acids Res.

