# Supplementary material for: Tracing the phylogenetic history of the Crl regulon through the Bacteria and Archaea genomes
Source: BMC Genomics. 2019 Apr 16;20:299. doi: 10.1186/s12864-019-5619-z (PMC6469107; doi:10.1186/s12864-019-5619-z)
Supplement: Supplementary file 1 — New members of the RpoS-Crl regulon. (DOCX 23 kb) [file 12864_2019_5619_MOESM1_ESM.docx]

Additional file 1

| **Gene** | **BNumber** | **Temperature at which it was determined as a member of RpoS Regulon** | **Evidence sigma and reference** | **Reference** | **Name of σS promoter predicted** [17] | **Absolute position of TSS** [17] |
| --- | --- | --- | --- | --- | --- | --- |
| *aat* | b0885 | Determined at 30ºC | GEA, IMP | [11] | *aat*p1  *aat*p2 | 926683  926725 |
| *accB* | b3255 | Determined at 30ºC | GEA, IMP | [11] |  |  |
| *accC* | b3256 | Determined at 30ºC | GEA, IMP | [11] |  |  |
| *acnB* | b0118 | Determined at 37ºC | GEA | [13] |  |  |
| *allR* | b0506 | Determined at 28ºC | MSI | [10] |  |  |
| *bfr* | b3336 | Determined at 30 and 37ºC | MSI, GEA, IMP | [40]^MSI^, [10]^GEA, IMP^ | *bfr*p2 | 3464771 |
| *bglG* | b3723 |  | MSI, IMP | [16] |  |  |
| *bioB* | b0775 | Determined at 37ºC | GEA | [13] |  |  |
| *cstA* | b0598 | Determined at 30ºC | GEA, IMP | [11] |  |  |
| *cysP* | b2425 | Determined at 30C | MSI | [10] |  |  |
| *djlC (ybeV)* | b0649 | Determined at 37ºC | MSI | [40]^MSI^, [10]^MSI^ |  |  |
| *flgM* | b1071 | Determined at 30 and 37ºC | GEA | [13] |  |  |
| *fliA* | b1922 | Determined at 37ºC | GEA | [13] |  |  |
| *fur* | b0683 |  | MSI, IMP | [12] |  |  |
| *gadW* | b3515 | Determined at 37ºC | MSI | [40]^MSI^, [10]^MSI^ |  |  |
| *glnH* | b0811 | Determined at 30ºC | GEA, IMP | [11] |  |  |
| *gltA* | b0720 | Determined at 30ºC | GEA, IMP | [11] |  |  |
| *grxB* | b1064 | Determined at 30ºC | GEA, IMP | [11] |  |  |
| *hdeD* | b3511 | Determined at 37ºC | MSI | [40]^MSI^, [11]^MSI^ |  |  |
| *hdhA* | b1619 | Determined at 30ºC | GEA, IMP | [11] |  |  |
| *malE* | b4034 | Determined at 30ºC | GEA, IMP | [11] |  |  |
| *msrB* | b1778 | Determined at 37ºC | GEA | [13] | *msrB*p3 | 1860691 |
| *ompT* | b0565 | Determined at 37ºC | GEA | [13] |  |  |
| *ompX* | b0814 | Determined at 30ºC | GEA, IMP | [11] |  |  |
| *paaA* | b1388 | Determined at 30ºC | MSI | [10] |  |  |
| *paaB* | b1389 | Determined at 30ºC | MSI | [10] |  |  |
| *paaD* | b1391 | Determined at 30ºC | MSI | [10] |  |  |
| *paaF* | b1393 | Determined at 30ºC | MSI | [10] |  |  |
| *paaH* | b1395 | Determined at 30ºC | MSI | [10] |  |  |
| *paaK* | b1398 | Determined at 30ºC | MSI | [10] |  |  |
| *ppa* | b4226 | Determined at 30ºC | GEA, IMP | [11] |  |  |
| *psiF* | b0384 | Determined at 37ºC | MSI | [40]^MSI^, [10]^MSI^ |  |  |
| *sdhC* | b0721 | Determined at 37ºC | GEA | [13] |  |  |
| *ssb* | b4059 | Determined at 30ºC | GEA, IMP | [11] |  |  |
| *tar* | b1886 | Determined at 37ºC | GEA | [13] |  |  |
| *tdcC* | b3116 | Determined at 28ºC | MSI | [10] |  |  |
| *tnaA* | b3708 | Determined at 30ºC | GEA, IMP | [11] |  |  |
| *ugpB* | b3453 | Determined at 30ºC | GEA, IMP | [11] |  |  |
| *uspA* | b3495 | Determined at 30ºC | GEA, IMP | [11] |  |  |
| *uspG (ybdQ)* | b0607 | Determined at 30ºC | GEA, IMP | [11] |  |  |
| *ybaY* | b0453 | Determined at 37ºC | MSI | [10] | *ybaY*p4 | 474528 |
| *ybgS* | b0753 | Determined at 37ºC | MSI | [10] | *ybgS*p4 | 784761 |
| *ycaC* | b0897 | Determined at 30 and 37ºC | MSI, GEA, IMP | [10]^MSI^, [11]^GEA, IMP^ |  |  |
| *yeaH* | b1784 | Determined at 37ºC | MSI | [10] |  |  |
| *yeeE* | b2013 | Determined at 30ºC | MSI | [10] |  |  |
| *yhjR* | b3535 | Determined at 37ºC | MSI | [10] | *yhjR*p9 | 3694418 |
| *yjbJ* | b4045 | Determined at 28ºC | MSI | [10] | *yjbJ*p3  *yjbJ*p4 | 4257187  4257201 |
| *ymdA* | b1044 | Determined at 28ºC | MSI | [10] | *ymdA*p2  *ymdA*p3  *ymdA*p4  *ymdA*p6 | 1104483  1104585  1104576  1104505 |
| *ymfE* | b1138 | Determined at 28ºC | MSI | [10] |  |  |

**New members of the RpoS regulon.**
