# Supplementary material for: Tracing the phylogenetic history of the Crl regulon through the Bacteria and Archaea genomes
Source: BMC Genomics. 2019 Apr 16;20:299. doi: 10.1186/s12864-019-5619-z (PMC6469107; doi:10.1186/s12864-019-5619-z)
Supplement: Supplementary file 2 — Number of regulated genes by different transcription factor in Crl Regulon. (DOCX 15 kb) [file 12864_2019_5619_MOESM2_ESM.docx]

Additional file 2

| **Number of regulated genes** | **Number of TFs** |
| --- | --- |
| 24 | 0 |
| 19 | 1 |
| 11 | 2 |
| 14 | 3 |
| 3 | 4 |
| 4 | 5 |
| 2 | 6 |
| 1 | 7 |
| 3 | 8 |
| 3 | 10 |
| 2 | 12 |

**Number of regulated genes by different transcription factor in Crl Regulon.**
